# Infant feeding practices among macrosomic infants: A prospective cohort study

**DOI:** 10.1111/mcn.13222

**Published:** 2021-06-01

**Authors:** Philippa Davie, Debra Bick, Dharmintra Pasupathy, Sam Norton, Joseph Chilcot

**Affiliations:** ^1^ Health Psychology Section, Department of Psychology, Institute of Psychiatry, Psychology and Neuroscience, Guy's Hospital King's College London London UK; ^2^ Warwick Clinical Trials Unit, Warwick Medical School University of Warwick Coventry UK; ^3^ Sydney Medical School, Westmead Clinical School University of Sydney Sydney New South Wales Australia; ^4^ Department of Women and Children's Health, Faculty of Life Sciences and Medicine, St Thomas' Hospital King's College London London UK

**Keywords:** birthweight, breastfeeding, feeding behaviour, infant, macrosomia, maternal health

## Abstract

The health benefits of breastfeeding are well recognised, but breastfeeding rates worldwide remain suboptimal. Breastfeeding outcomes have yet to be explored among women who give birth to macrosomic (birthweight ≥4000 g) infants, a cohort for whom the benefits of breastfeeding may be particularly valuable, offering protection against later‐life morbidity associated with macrosomia. This longitudinal prospective cohort study aimed to identify whether women who give birth to macrosomic infants are at greater risk of breastfeeding non‐initiation or exclusive breastfeeding (EBF) cessation. A total of 328 women in their third trimester were recruited from hospital and community settings and followed to 4 months post‐partum. Women gave birth to 104 macrosomic and 224 non‐macrosomic (<4000 g) infants between 2018 and 2020. Longitudinal logistic regression models calculated odds ratios (ORs) and 95% confidence intervals (CIs) to assess likelihood of EBF at four timepoints post‐partum (birth, 2 weeks, 8 weeks, and 4 months) between women who gave birth to macrosomic and non‐macrosomic infants, adjusted for maternal risk (obesity and/or diabetes), ethnicity and mode of birth. Macrosomic infants were more likely to be exclusively breastfed at birth and 2 weeks post‐partum than non‐macrosomic infants with adjusted OR = 1.94 (95% CI: 0.90, 4.18; *p* = 0.089) and 2.13 (95% CI: 1.11, 4.06; *p* = 0.022), respectively. There were no statistically significant associations between macrosomia and EBF at 8 weeks or 4 months post‐partum. Macrosomia may act as a protective factor against early formula‐milk supplementation, increasing the likelihood of EBF in the early post‐partum period, but rates of exclusive breastfeeding continued to decline over the first 4 months post‐partum.

**Key messages**

- Breastfeeding outcomes are not routinely explored among infants born with a macrosomic birthweight (≥4000 g).
- A longitudinal cohort study identified macrosomic infants were more likely to be exclusively breastfed at birth and 2 weeks post‐partum.
- Exclusive breastfeeding rates were not significantly different between macrosomic and non‐macrosomic infants at 8 weeks and 4 months post‐partum.
- Macrosomia may offer some protection against breastfeeding latch and lactation difficulties in the first 2 weeks post‐partum; however, this effect appears to be lost over time.
- Women who give birth to healthy‐term macrosomic infants are unlikely to require specialist breastfeeding counselling/support that differs from gold‐standard post‐natal care.

## 1 INTRODUCTION

Macrosomia is associated with adverse perinatal outcomes (e.g., shoulder dystocia, brachial plexus injury and low APGAR scores) and maternal morbidity (e.g., post‐partum haemorrhage, caesarean section and obstetric anal sphincter injury) (Beta et al., 2019; Boulet et al., 2003; Jolly et al., 2003). Definitions of macrosomia vary between ≥4000 g and ≥4500 g, based on thresholds associated with increased maternal and neonatal complications (Boulet et al., 2003). Fetal overgrowth is also defined according to large for gestational age (LGA): birthweight >90th or 95th centile adjusted for gestational age and sex (Vieira et al., 2020). While there is overlap between the definitions, there is no universal consensus on a single criteria. Macrosomic infants represent a growing proportion of infants in the United Kingdom, which can be partially attributed to increasing prevalence of maternal obesity and diabetes (Henriksen, 2008; Poston, 2012). The health benefits associated with breastfeeding are well‐substantiated (Victora et al., 2016) and may provide an early‐life intervention to curb the intergenerational risk cycle of macrosomia, obesity and diabetes (Catalano, 2003; Poston, 2012). However, breastfeeding is not routinely reported as a perinatal health outcome for macrosomic infants. Limited evidence available for the association between macrosomia and breastfeeding (exclusivity and duration) is complex and contradictory with studies suggesting positive (Jolly et al., 2003; Leonard & Rasmussen, 2011), negative (Lande et al., 2005) and no associations (Oddy et al., 2006). To the best of our knowledge, no studies to date have systematically examined whether macrosomic infants are at increased odds of not receiving breast milk or early breastfeeding cessation within 6 months of birth.

Evidence suggests macrosomia is associated with increased risk of diabetes, cardiovascular disease and obesity in childhood and later life (Monasta et al., 2010; Whincup et al., 2008). Sustained, exclusive breastfeeding is recognised to attenuate the risk of such morbidities (Horta et al., 2015; Horta & Victora, 2013; Victora et al., 2016). For macrosomic infants, the risk of obesity and associated morbidities may be attenuated by optimal feeding practices early on. Exclusive formula‐feeding is associated with rapid weight gain in early infancy (Griffiths et al., 2009). Such rapid growth in the first 3 months of life can significantly increase the likelihood of obesity in childhood (Zhang et al., 2013). If macrosomic infants are predominantly or exclusively formula‐fed in the first 6 months, high birthweight trajectories may ‘track’ into infancy and childhood. For example, a longitudinal study indicated no significant differences in body mass index (BMI) at age 3 between infants born LGA and average‐for‐gestational‐age (AGA), so long as LGA infants were breastfed for at least 12 months; LGA infants who were breastfed for shorter durations (<12 months) remained significantly larger at age three compared with infants born AGA, highlighting the potential moderating impact of breastfeeding in the association between larger birthweight and later‐life health status (Çamurdan et al., 2011). Breastfeeding is likely to provide valuable protection for macrosomic infants. Understanding feeding practices in this cohort may provide direction for health interventions aimed at supporting the preconception, pregnancy and post‐partum health of women and their infants. This study therefore aimed to identify whether macrosomic infants are more or less likely to be exclusively breastfed from birth up to 4 months post‐partum. The study explores the hypothesis that macrosomia (birthweight ≥4000 g) is associated with increased or decreased likelihood of EBF.

## 2 METHODOLOGY

### 2.1 Ethical considerations

Participating women provided fully informed written consent prior to study commencement. This study was approved by a regional Research Ethics Committee (REC) on 1 August 2018 (Ref:18/LO/0740) overseen by the Health Research Authority. All data were handled according to Good Clinical Practice guidelines.

### 2.2 Study population

**FIGURE 1:**
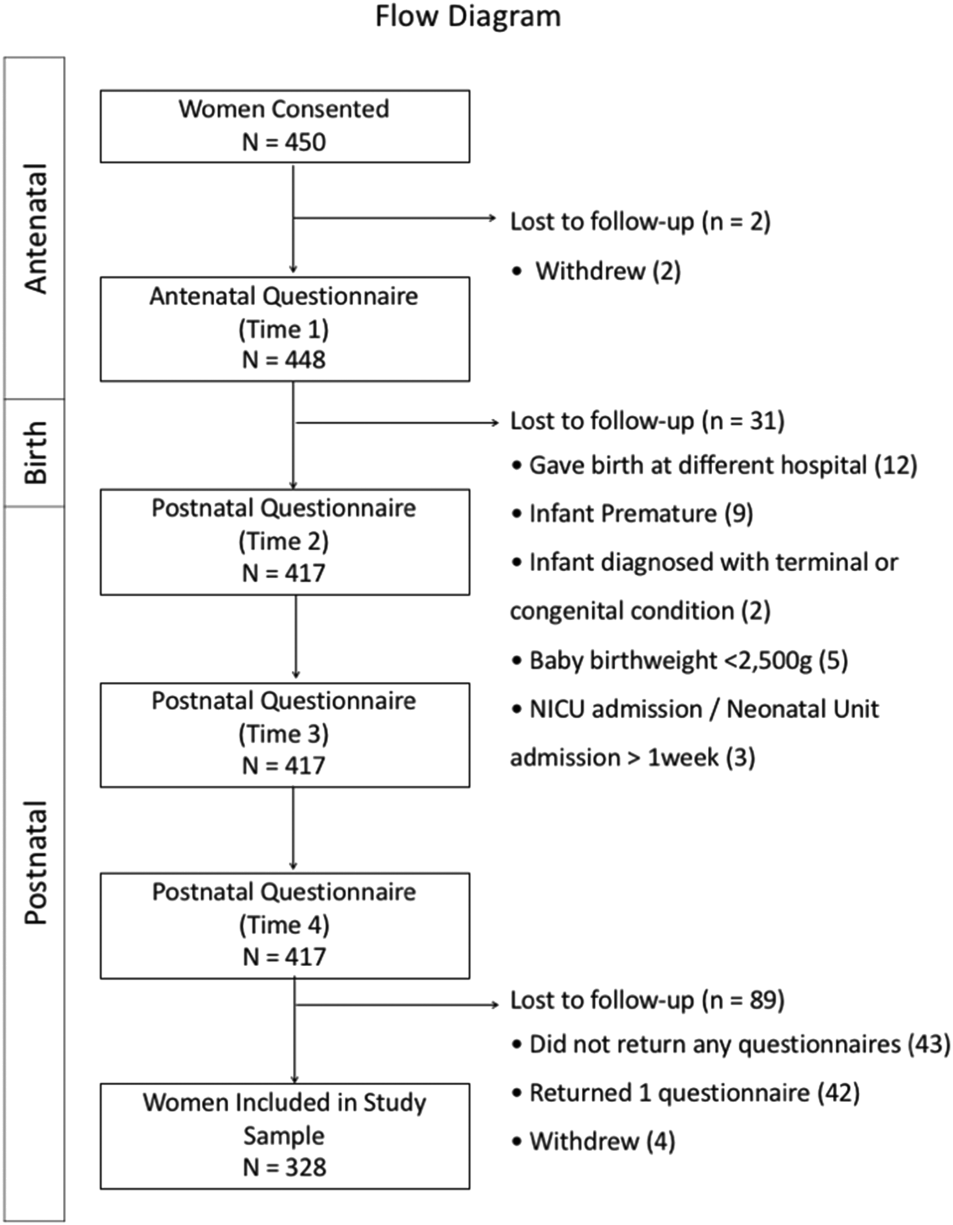
Flow diagram of participants included in cohort study. Reasons women withdrew from the study included moved away from study location (2), excessive commitments (1) and no reasons provided (3)

A total of 450 women were recruited into the study, with 328 included in final analyses. A flowchart of women participating in the study and inclusion of study participants in the final sample is presented in Figure 1.

To be included in the study, women had to be aged ≥18 years, give birth to a healthy, term (≥37^+0^ weeks) singleton baby. Women diagnosed with cardiac, renal, autoimmune or malignant disease and women with mental health conditions requiring specialist care pathways were not eligible for recruitment. Infants born with congenital conditions or morbidity, admitted to neonatal intensive (NICU) or high dependency care or special care baby unit (≥1 week) were excluded from the study. Questionnaire data had to be returned at least twice for women to be included in final study sample.

### 2.3 Procedure

Women were recruited from hospital and community sites served by one South London NHS Foundation Trust using one of two sampling methods. Women were either recruited antenatally (≥28^+0^ weeks gestation) opportunistically according to eligibility criteria, or invited to take part purposively post‐natally (≤14 days post‐partum) if they met full eligibility criteria and gave birth to a macrosomic infant. This aimed to include a higher proportion of macrosomic infants in the study than relying on opportunistic sampling alone, to allow sufficiently powered statistical analyses.

Following consent to participate, postal and/or online questionnaires were sent to women to complete during pregnancy (if recruited antenatally), at 2 weeks and 4 months post‐partum. Questionnaire data at 8 weeks were collected via telephone calls made between 6 and 8 weeks post‐partum. Pregnancy and infant related medical information were collected via medical records at time of birth.

### 2.4 Patient and public involvement (PPI)

An independent PPI group reviewed the study materials (information sheet, consent form and questionnaires) via email before study commencement to provide feedback on clarity and suitability. The group consisted of five multiparous women who were currently pregnant and had previous personal experience of childbirth, maternity care and infant feeding; these women did not take part in the research study. No amendments were recommended to the items used to collect sociodemographic, clinical, infant or infant feeding data. Feedback from women found the questionnaires were easily understood and simple to complete.

When the study was closed and all women had completed follow‐up (September 2020), a research dissemination event was held to report main outcomes to all participating women. Written and verbal feedback was positive: women reported their experiences were represented in the interpretations of the data available. During the study, some women reported concern about being purposively invited based on infant macrosomia.

### 2.5 Study variables

Data were collected via self‐report questionnaires and medical records. Questionnaire booklets were designed by the study authors and collected data about women's sociodemographic characteristics, clinical risk, infant characteristics and infant feeding practices. The questionnaires took no longer than 15 min to complete at each timepoint and could be completed online or via paper.

At baseline, women self‐reported sociodemographic characteristics: age (years), ethnicity (Asian, Black, Hispanic, Mixed‐ethnicity, White), country of birth (UK vs. not UK), education (highest qualification obtained), marital status (married/de facto vs. single/separated) and parity (multiparous vs. primiparous). Postcode data provided were used as a proxy indicator of socioeconomic status according to Index of Multiple Deprivation (Office of National Statistics, 2019) ranked in deciles (0 = most deprived to 10 = least deprived). Women's risk status according to pregnancy booking BMI (BMI ≥ 30 kg/m^2^ vs. BMI < 30 kg/m^2^) and diabetic status (Type‐I, Type‐II and gestational diabetes [GDM]) were recorded from self‐report and verified via medical records. Women were classed as high‐risk if they had a BMI ≥ 30 kg/m^2^ or a diagnosis of diabetes. Low‐risk women had BMI < 30kgm/^2^ and no diagnosis of diabetes during pregnancy.

Infant characteristics were collected via medical records at birth and included infant sex, birthweight (g), gestational age, NICU admission (yes or no) and mode of birth: a categorical outcome (vaginal unassisted; vaginal assisted, i.e., with ventouse or forceps; planned caesarean; unplanned or emergency caesarean). The main exposure was infant macrosomia at birth defined as ≥4000 g or <4000 g (non‐macrosomic).

### 2.6 Main outcome

Breastfeeding practices at each follow‐up timepoint were captured using a self‐report questionnaire. A proportionate measure of infant feeding (Davie et al., 2018) rating feeding practices in the past 48 h on an 11‐point discrete scale ranging from 0% (exclusively formula‐fed) to 100% (exclusively breastfed) was used. The scale has not been tested for psychometric validity or reliability, but assessments of face validity during development found the scale was simple, clear and easy to understand. The scale has been recommended for use in infant feeding research as an alternative to traditional categorical measurements of infant feeding practices (Labbok & Krasovec, 1990; WHO, 2003) to capture behaviour on a gradient before collapsing data into categories. Infant feeding practices in the first 48 h post‐partum were recalled during completion of the first post‐natal questionnaire. Reported feeding practices were then categorised according to Interagency Group for Action on Breastfeeding (Labbok & Krasovec, 1990): exclusive breastfeeding (100% breast milk), partial breastfeeding high (>50%–90% breast milk), partial breastfeeding low (20%–≤50% breast milk), exclusive formula‐feeding/tokenistic breastfeeding (≤10% breast milk). Main outcome data compared likelihood of exclusive breastfeeding (EBF) over time depending on macrosomic status at birth.

### 2.7 Statistical methods

Associations between individual sociodemographic and clinical covariates and macrosomia were first examined using independent samples t‐test (continuous normally distributed data; Mann–Whitney *U* test for non‐parametric data) and Pearson's chi‐square test (for categorical data). These data are presented in Table 1.

**TABLE 1:** Sociodemographic and clinical characteristics of women and infants in study sample

	Non‐macrosomic (<4000g)	Macrosomic (≥4000g)	Statistic	*p* value (α = 0.05)
	(*N* = 224)	(*N* = 104)		
Risk			*χ*^2^ = 10.63	0.001*
Low	151 (67.41%)	88 (84.62%)		
High	73 (32.59%)	16 (15.38%)		
Diabetes			*χ*^2^ = 22.70	<0.001*
No DM	164 (72.31%)	95 (91.35%)		
GDM	56 (25.0%)	4 (3.85%)		
Type‐1	4 (1.79%)	5 (4.81%)		
BMI category			*χ*^2^ = 1.36	0.714
BMI ≤24.99	141 (62.95%)	61 (58.65%)		
BMI 25.0–29.99	51 (22.77%)	26 (25.0%)		
BMI ≥30.0	29 (12.95%)	10 (9.62%)		
BMI (SD)	24.84 (5.03)	25.01 (5.19)	t = 0.27	0.786
Age (SD)	34.99 (4.54)	35.28 (4.05)	t = 0.55	0.584
SES^a^	4 (3–6)	4 (3–7)	*z* = 1.76	0.076
Ethnicity			*χ*^2^ = 13.34	0.020*
Asian	24 (10.71%)	3 (2.88%)		
Black	24 (10.71%)	6 (5.77%)		
Hispanic	3 (1.34%)	4 (3.85%)		
Mixed race	15 (6.70%)	5 (4.81%)		
White	126 (56.25%)	77 (74.04%)		
UK born			*χ*^2^ = 1.26	0.263
Yes	94 (41.96%)	55 (52.88%)		
Marital status			*χ*^2^ = 6.79	0.147
Married/CP/de facto	181 (80.80%)	92 (88.47%)		
Single/separated	11 (4.91%)	4 (3.84%)		
Education			*χ*^2^ = 6.74	0.081
Secondary	1 (0.45%)	3 (2.88%)		
College	15 (6.70%)	2 (1.92%)		
University (UG)	63 (28.13%)	31 (29.81%)		
University (PG)	113 (50.45%)	58 (55.77%)		
Parity			*χ*^2^ = 1.28	0.258
Primiparous	111 (49.55%)	49 (47.12%)		
Baby sex			*χ*^2^ = 1.35	0.246
Boy	116 (51.79%)	61 (58.65%)		
Gestation at birth (weeks)	39 + 4 (1.23)	40 + 2 (1.14)	t = 4.98	<0.001*
Baby size			*χ*^2^ = 142.44	<0.001*
SGA	18 (8.04%)	0 (0%)		
AGA	200 (89.29%)	42 (40.38%)		
LGA	6 (2.68%)	62 (59.62%)		
Centile^b^	44.83 (25.97)	90.41 (8.27)	t = 17.48	<0.001*
Birthweight (g)	3343 (342)	4230 (184)	t = 24.78	<0.001*
Birth mode			*χ*^2^ = 6.98	0.073
Vaginal unassisted	93 (41.52%)	34 (32.69%)		
Vaginal assisted	45 (20.09%)	17 (16.35%)		
Caesarean (planned)	48 (21.43%)	22 (21.15%)		
Caesarean (emergency)	36 (16.07%)	29 (27.88%)		

Abbreviation: CP, civil partnership; DM, diabetes mellitus; SES, socio‐economic status.

^a^ Median rank and interquartile range (IQR) reported.

^b^ Centile according to WHO‐UK growth centiles (Royal College of Paediatrics and Child Health [RCPCH], 2013) (preterm adjusted).

* Significant at the α = 0.05 level.

To test main outcome associations, logistic regression models were used to estimate odds ratios (ORs) with 95% confidence intervals (CIs) for the association between macrosomic and likelihood of EBF over four timepoints, before and after adjustment for possible confounding effects of risk (high or low), ethnicity (White or Black or minority ethnic) and mode of birth (vaginal or caesarean). Confounders were considered based on existing evidence for factors associated with both macrosomia and breastfeeding practices (Ehrenberg et al., 2004; Flores et al., 2018; McAndrew et al., 2012; Prior et al., 2012). To understand potential confounding associations in this sample, unadjusted logistic regression models tested independent associations between maternal risk status, ethnicity, mode of birth and likelihood (ORs 95% CIs) of infant macrosomia. Logistic regression models were also used to estimate ORs (95% CIs) for the likelihood of breastfeeding initiation (starting breastfeeding within 48 h of birth) dependent on infant macrosomia. Statistical significance level (α) was set to 0.05 with 95% CIs. ORs (95% CIs) for breastfeeding behaviour at each time point were estimated using a logistic generalised estimating equation (GEE) approach, returning population‐averaged ORs that have the same interpretation as standard logistic regression models. Analyses were conducted using Stata (v16.1) (StataCorp, 2019).

A power calculation was conducted to estimate the required sample size to detect differences in breastfeeding rates between macrosomic and non‐macrosomic infants using logistic regression. The estimate used a weighted sample methodology, assuming prevalence of macrosomia in the United Kingdom is 9% (NHS, 2019), setting power at 80% and alpha at 5% (α = 0.05 level of significance). Calculations estimated a total of 315 women (including at least 12% macrosomic infants) were needed in analyses to detect a difference of OR = 2.50 for EBF at 4 months between women with macrosomic and non‐macrosomic infants.

### 2.8 Missing data

To identify potential sources of bias introduced to data available, independent samples t‐test (Mann–Whitney *U* test for non‐parametric data) and Pearson's chi‐square test (for categorical data) compared baseline characteristics between women who completed and did not complete (completed ≤1 questionnaire) the study. This missing data analysis is presented in Table S1. A significantly higher proportion of non‐completers were high‐risk (45.88% vs. 27.13%) and had GDM (31.76% vs. 21.04%) and obesity (28.24% vs. 11.89%). Non‐completers were also significantly younger (33.38 years vs. 35.08 years), had higher BMI (26.91 kg/m^2^ vs. 24.89 kg/m^2^), and were from more deprived areas (mdn = 4 [2.5–5] vs. 4 [3–6]). A significantly higher proportion of completers gave birth to macrosomic infants (31.61% vs. 15.48%).

To identify potential sources of bias within the final study sample, logistic regression models (ORs and 95% CIs) were used to observe associations between response‐level missing data (whether women provided data at that timepoint or not) and sociodemographic and clinical characteristics. Missing data were found to be missing conditional on known variables within the dataset. Given these observations, the impact of missing data on study estimates was accounted for in two ways: (i) breastfeeding rates were estimated using a model assuming *missing at random* conditional on mother's age, ethnicity, risk status and mode of birth and (ii) logistic regression models were weighted by the inverse probability of non‐response, which was conditioned on the variables listed above.

## 3 RESULTS

### 3.1 Descriptive data

Full sociodemographic and clinical characteristics are presented in Table 1. A total of 89 women (27.13%) were high‐risk: obesity (*n* = 20); GDM (*n* = 42); comorbid obesity and GDM (*n* = 18); Type‐I DM (*n* = 8); comorbid obesity and Type‐I DM (*n* = 1).

Women with high‐risk were significantly less likely to give birth to macrosomic infants than low‐risk women (OR = 0.38; 95% CI: 0.21, 0.69; *p* = 0.001). Macrosomic infants had significantly increased risk of being born via emergency caesarean section (OR = 2.20; 95% CI: 1.18, 4.13; *p* = 0.014) than vaginally (unassisted) compared with non‐macrosomic infants. Black women and women from minority ethnic backgrounds were significantly less likely to give birth to macrosomic infants (OR = 0.45; 95% CI: 0.25, 0.81; *p* = 0.008) compared with White women. Average pregnancy gestation was significantly longer among women who gave birth to macrosomic infants (40^+2^ vs. 39^+4^, *p* < 0.001).

### 3.2 Main outcome: breastfeeding practices

In the total sample, 94.76% of women reported initiating breastfeeding within 48‐hours of birth (81.47% exclusively, 13.29% partially). However, the proportion of women breastfeeding exclusively fell across the post‐partum period to 74.34% at 2 weeks, 67.08% at 8 weeks and 63.44% at 4 months as shown in Table 2.

**TABLE 2:** Comparison of breastfeeding practices and breastfeeding exclusivity among macrosomic and non‐macrosomic infants over time

	48 h		2 weeks		8 weeks		4 months	
	<4000 g	≥4000 g	<4000 g	≥4000 g	<4000 g	≥4000 g	<4000 g	≥4000 g
Total (*N*)	191	95	171	94	170	73	187	92
Formula‐feeding	14 (7.33%)	1 (1.05%)	9 (5.26%)	1 (1.06%)	11 (6.47%)	8 (10.96%)	28 (14.97%)	10 (10.87%)
Partial BF (low)	12 (6.28%)	4 (4.21%)	14 (8.19%)	8 (8.51%)	16 (9.41%)	3 (4.1%)	11 (5.88%)	3 (3.26%)
Partial BF (high)	16 (8.38%)	6 (6.32%)	29 (16.96%)	7 (7.45%)	28 (16.47%)	14 (19.18%)	31 (16.58%)	19 (20.65%)
Exclusive BF	149 (78.01%)	84 (88.42%)	119 (69.59%)	78 (82.98%)	115 (68.82%)	48 (65.75%)	117 (62.57%)	60 (65.22%)
BF exclusivity^a^ (%)								
Mean (SD)	9.01 (2.56)		8.82 (2.55)		8.42 (2.97)		8.01 (3.49)	
Macrosomic vs. non‐macrosomic	8.75 (2.89)	9.54 (1.60)	8.57 (2.79)	9.26 (1.96)	8.46 (2.88)	8.33 (3.18)	7.85 (3.62)	8.35 (3.19)
*p* value	0.014*		0.037*		0.755		0.261	

*Note*: Infants had an average age of 23.3 days (SD = 13.38) when the first questionnaire was returned (sent out at 2 weeks post‐partum), 56.8 days when telephone questionnaires were completed between 6 and 8 weeks, and 128.6 days (SD = 13.56) at final questionnaire completion (sent out at 4 months post‐partum).

^a^ Exclusivity is rated on a proportionate scale of infant feeding where 0 = 0% breastfeeding (100% formula‐feeding) and 10 = 100%, that is, exclusive breastfeeding.

* Significant at the α = 0.05 level.

In total, 98.95% of macrosomic infants received breast milk (exclusively or partially) in the first 48 h after birth, compared with 92.67% of non‐macrosomic infants. Adjusted logistic GEE models suggest women with macrosomic infants were considerably more likely to initiate breastfeeding (adj. OR = 4.98; 95% CI: 0.70, 35.26; *p* = 0.108); however, given the large uncertainty in the estimates, the null hypothesis cannot be rejected with confidence.

**FIGURE 2:**
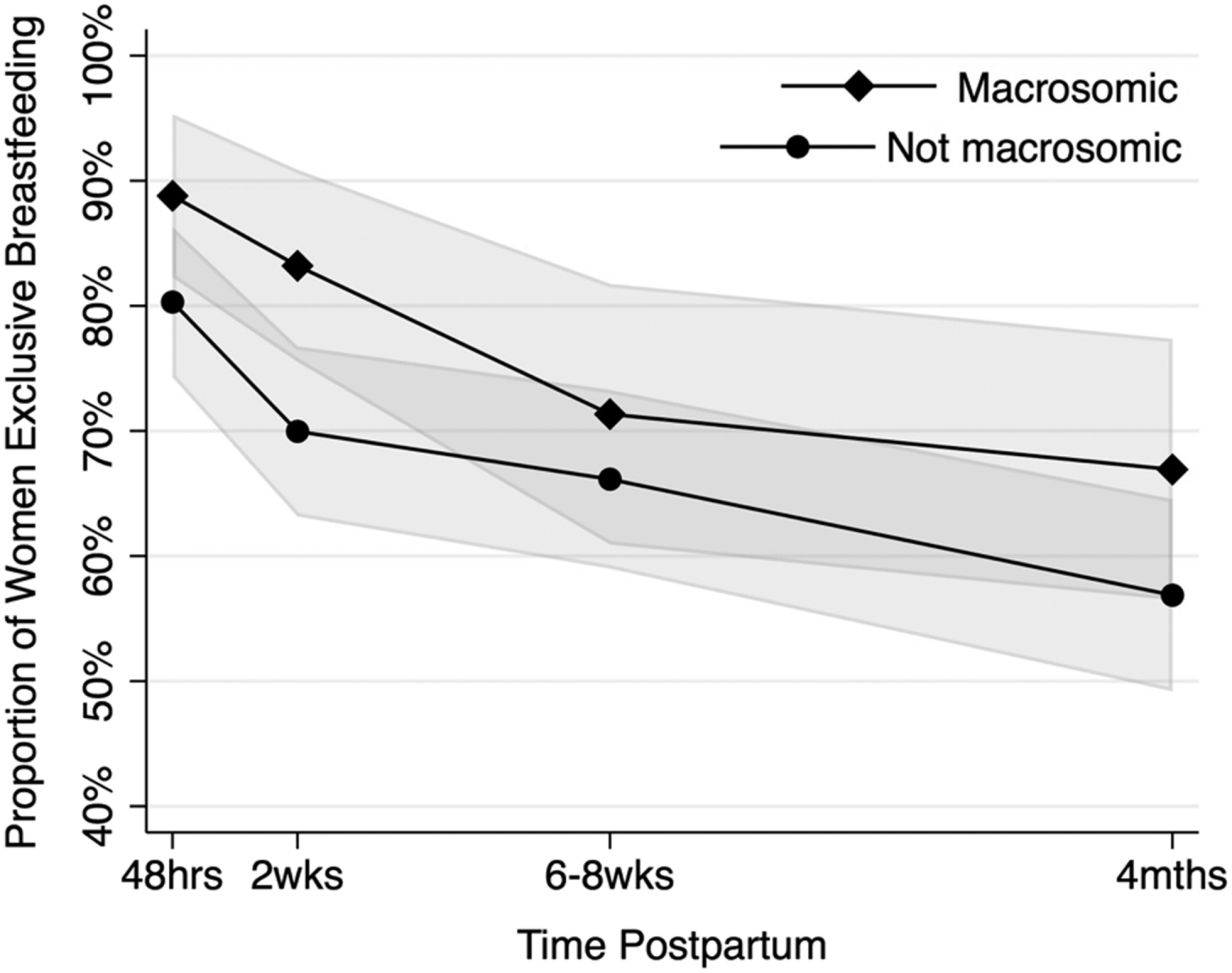
Profile plot estimating proportion of women exclusively breastfeeding over time adjusted for maternal risk, ethnicity and mode of birth. *Note*: Shaded areas show 95% CIs around each estimated proportion using delta method. This method has lower precision than significance tests for ORs so overlap in 95% CIs should not be interpreted as non‐significant associations (failing to reject null hypothesis)

The proportion of women breastfeeding exclusively across macrocosmic and non‐macrocosmic infant groups is displayed in Figure 2. At each timepoint, rates were higher for those with macrocosmic infants. As presented in Table 3, unadjusted logistic GEE regression models indicate macrosomic infants were significantly more likely to be EBF at birth and 2 weeks post‐partum than non‐macrosomic infants with OR = 2.05 (95% CI: 1.03, 4.10) and OR = 1.95 (95% CI: 1.09, 3.48), respectively. Adjusted logistic GEE regression models indicate macrosomic infants were 1.94 times (95% CI: 0.90, 4.18) more likely to be EBF in the first 48 h after birth than non‐macrosomic infants, but the association did not reach statistical significance. At 2 weeks, macrosomic infants were significantly more likely to be EBF (adjusted OR = 2.13; 95% CI: 1.11, 4.06). Small associations were observed between macrosomia and EBF at 8 weeks and 4 months post‐partum suggesting macrosomic infants were more likely to be EBF, but associations did not reach statistical significance in unadjusted or adjusted models (see Table 3).

**TABLE 3:** Odds ratios and 95% confidence intervals derived from logistic regression models observing associations between macrosomia and exclusive breastfeeding at each timepoint

Timepoint	Unadjusted			Adjusted^a^		
	ORs	95% CI	p	ORs	95% CI	p
48 h	2.05	1.03, 4.10	0.042*	1.94	0.90, 4.18	0.089
2 weeks	1.95	1.09, 3.48	0.025*	2.13	1.11, 4.06	0.022*
8 weeks	1.20	0.70, 2.06	0.517	1.27	0.69, 2.35	0.437
4 months	1.27	0.77, 2.10	0.342	1.53	0.86, 2.73	0.145

^a^ Adjusted for maternal risk status, ethnicity and mode of birth.

## 4 DISCUSSION

Data from this prospective longitudinal cohort study suggest macrosomic infants are more likely to be exclusively breastfed in the early post‐natal period than non‐macrosomic infants, but the impact of macrosomia is likely to be less important over time up to 4 months post‐partum. It is unclear whether macrosomic infants are at increased risk of breastfeeding non‐initiation. Given power to detect significant differences between groups is low, data are interpreted as indicating women with macrosomic infants have a moderately increased likelihood of EBF in the first 2 weeks after birth, with confidence intervals that exclude the null hypothesis. At 8 weeks and 4 months, ORs and confidence intervals cannot reject the null hypothesis, which states there is no association between macrosomia and likelihood of EBF. The difference in likelihood of EBF between macrosomic and non‐macrosomic infants is largest at 2 weeks and smallest at 8 weeks, where adjusted absolute differences were 13.2% and 5.2% respectively (see Table 2).

Breastfeeding initiation rates (counted as putting baby to the breast even once) are high across settings in the United Kingdom (McAndrew et al., 2012), which correspond with findings of this study where initiation was reported to be over 90%. The relatively low frequency of women who did not initiate breastfeeding (either partially or exclusively) reduces power to detect statistically significant differences in adjusted models, which may partially explain the uncertainty of estimates in this study. Findings should be interpreted with the understanding this cohort included healthy, full‐term infants, meaning patterns of breastfeeding may be different among macrosomic infants requiring specialist care pathways in the first days of life. These interpretations are consistent with the limited evidence available for associations between macrosomia and breastfeeding.

Findings of this study complement previous research suggesting women who gave birth to macrosomic infants were more likely to initiate breastfeeding (Ayukarningsih & Dwinanda, 2015; Jolly et al., 2003) and that breastfeeding initiation was high independent of macrosomia (Oddy et al., 2006). Data from a Zimbabwe national health survey (Mukora‐Mutseyekwa et al., 2019) suggested macrosomic birthweight (≥4000 g) was significantly associated with delayed early initiation (≤1 h) of breastfeeding after adjustment for sociodemographic factors. The study did not control for birth‐related confounders but noted higher birthweight infants were more likely to be separated from their mothers after birth due to intrapartum intervention, which may have contributed to lower rates of early initiation. Mode of birth may also partially explain adjusted associations observed at birth in this study. Macrosomic infants were significantly more likely to be born via emergency caesarean section, which can impact the establishment of EBF and initiation rates (Prior et al., 2012).

Inverse associations between maternal risk (i.e., obesity and/or diabetes) and likelihood of macrosomia are unexpected given evidence available (Boulet et al., 2003; Ehrenberg et al., 2004; Jolly et al., 2003; Poston, 2012; Vieira et al., 2020). This may be partially attributed to both sampling method and local clinical care practices. Women with a macrosomic infant recruited purposively post‐natally were significantly more likely to be low‐risk, thus increasing the proportion of macrosomic infants born to low‐risk women in the study sample through selection bias. In addition, clinical care available at the study location included increased antenatal monitoring of women with obesity and diabetes (T1, T2 and GDM), designed to decrease the likelihood that high‐risk women give birth to macrosomic and/or LGA infants (NICE, 2015; NICE, 2019). Therefore, low‐risk women were overrepresented among women who give birth to macrosomic infants.

At 2 weeks post‐partum, macrosomic infants were significantly more likely to be exclusively breastfed (i.e., not supplemented with formula‐milk) than non‐macrosomic infants, suggesting a larger infant birthweight may facilitate breastfeeding. It is possible macrosomic infants may have larger mouths, increased strength or head control movement, which could facilitate the physiology of breastfeeding latching and reduce the likelihood of nipple pain and trauma; one of the most common reasons for early breastfeeding cessation (McAndrew et al., 2012; McClellan et al., 2012). By latching effectively, infants stimulate milk production and facilitate the physiological demand and supply processes involved in lactation (Wambach & Genna, 2019). This could reduce the need (objective or subjective) for supplementation with formula‐milk and therefore increase the likelihood that women sustain EBF. A recent qualitative study comparing women's experiences of breastfeeding macrosomic and non‐macrosomic infants suggested a larger infant birthweight may protect against breastfeeding difficulties in the early post‐partum period and attenuate maternal concerns (Davie et al., 2021).

While macrosomia may offer some protection against breastfeeding latch and lactation difficulties in the first 2 weeks post‐partum (resulting in higher rates of exclusive breastfeeding), any protective effect offered by a larger birthweight appears to be lost by 8 weeks post‐partum. Women with macrosomic infants stopped breastfeeding at a faster rate between 2 and 8 weeks post‐partum than women with non‐macrosomic infants. In the United Kingdom, infant health check‐ups are offered routinely between 6 and 8 weeks post‐partum and include infant weighing. Clinical care and feeding advice offered at this timepoint may influence this attrition rate; however, factors explored in this study were not able to offer any further insight into why attrition may be greater at this timepoint among women with macrosomic infants. Future research exploring patterns of infant feeding in the first 8 weeks, including experiences of routine infant health checks, among women who give birth to macrosomic infants would be beneficial. As attrition of exclusive breastfeeding is greater among women with macrosomic infants, additional lactation support during these post‐natal weeks would be beneficial. However, breastfeeding rates in both groups decline rapidly within the first 8 weeks post‐partum highlighting the recognised need for improved and prolonged lactation support for all women, regardless of infant birthweight.

Although the evidence is currently not conclusive (Monasta et al., 2010; Poston, 2012), being macrosomic at birth does not guarantee an infant will be heavier or LGA throughout infancy. For example, guidance available (NICE, 2017) categorises a drop of three growth percentiles as a threshold for concern for infants born LGA (>90th centile), compared with two centiles for infants born AGA (≤90th centile) highlighting expected growth that regresses towards the population average. Given the rapid changing growth and weight of a newborn in the first weeks of life, it is unclear whether a larger infant birthweight will have a sustained and independent impact on EBF practices over the post‐natal period, meaning associations observed in this study are plausible.

### 4.1 Strengths and limitations

To the best of our knowledge, this is one of the first studies to systematically investigate associations between larger infant birthweight and breastfeeding practices over time controlling for maternal risk status (i.e., obesity and diabetes).

Previous research reporting associations between larger infant birthweight (macrosomia or LGA) and infant feeding practices has not been designed to compare practices between macrosomic and non‐macrosomic infants (Leonard & Rasmussen, 2011; Oddy et al., 2006), are retrospective questionnaire studies (Lande et al., 2005), or only reported infant feeding outcomes at one time (Jolly et al., 2003; Mukora‐Mutseyekwa et al., 2019). This study benefits from a prospective longitudinal design, including a higher proportion of macrosomic infants (46.43%) than would be observed under opportunity sampling in the target population (NHS, 2019), increasing power to detect significant differences in EBF rates over time. However, given the study sample size and low frequency of women who did not initiate breastfeeding, power in the study remained low. Low statistical power is a notable limitation of the evidence provided in this study. Associations between maternal BMI, diabetic status, macrosomia, and breastfeeding practices are complex and interacting (Poston, 2012), making it difficult to quantify independent effects (Ehrenberg et al., 2004). The proportion of high‐risk women included in the sample meant it was not possible to reliably compare feeding practices between high‐risk and low‐risk women. However, controlling for risk as a confounding factor helps to observe potentially independent effects of macrosomic birthweight.

Collection of clinical data (e.g., birthweight, mode of birth, prematurity and maternal risk status) from medical records was implemented to avoid recall bias and improve accuracy of data. However, data on infant feeding practices were reliant on self‐report questionnaire measures, which are susceptible to response bias and inaccuracies in recall. Implementing a 48‐h recall method advocated by the measurement, and comparable with WHO (2003) recommendations, aimed to reduce inaccuracies, but it is recognised that recall method may not capture true breastfeeding patterns over time. Women may overestimate breastfeeding exclusivity either inadvertently or out of desire to report their behaviour as adherent to clinical recommendations for infant feeding, which introduces response bias in estimates observed.

An attrition rate of 20.22% from baseline to final follow‐up at 4 months was observed, with a notable selection bias introduced among completing women. Women with high‐risk (GDM and higher BMI, particularly obesity), younger age and living in more socioeconomically deprived areas were significantly more likely to drop out. Each of the characteristics are typically associated with significantly decreased likelihood of breastfeeding initiation and shorter breastfeeding durations (exclusive or non‐exclusive) (Finkelstein et al., 2013; Flores et al., 2018; McAndrew et al., 2012). Is it therefore likely additional response bias was introduced, with breastfeeding women more likely to continue the study than women who ceased breastfeeding. This is plausible particularly when the relatively high rates of EBF (63.44%) at 4 months in the study population compared with national estimates (12%; McAndrew et al., 2012) are considered. Demographic biases in the study in terms of ethnicity, educational attainment and marital status (each associated with breastfeeding behaviour) further limit the generalisability of findings to more diverse populations.

The generalisation of study findings are also limited by the exclusion of infants admitted to NICU or cared for in special care units (≥1 week). Due to increased risk of adverse perinatal outcomes, macrosomic infants are more likely to require admission to neonatal care units after birth (Beta et al., 2019; Boulet et al., 2003; Jolly et al., 2003), which presents unique barriers to breastfeeding initiation and establishment (Spatz, 2004). Associations observed in this study may not been observed among macrosomic infants admitted to NICU.

## 5 CONCLUSION

This cohort study observed increased rates of EBF among macrosomic infants in the early post‐natal period; however, trends in EBF decline over time independent of macrosomia. Given the increased rates of EBF observed, women who give birth to healthy, term macrosomic infants are unlikely to require specialist care or tailored breastfeeding support that differs from routine post‐natal care. In order to effectively protect and support breastfeeding, post‐natal care should be consistent and predictable, tailored to women's individual care needs, and include access to specialist lactation support and advice across the post‐partum period and health care settings (McFadden et al., 2017; Sinha et al., 2015; WHO, 2013; WHO, 2018).

Findings from this study are limited to women who give birth to healthy, full‐term macrosomic infants. This study was not able to disentangle potential interaction effects between macrosomia, maternal risk and adverse perinatal outcomes on likelihood of breastfeeding. Given the complex physiological, clinical and psychosocial associations between maternal obesity, diabetes, fetal macrosomia, perinatal morbidity, breastfeeding and long‐term health, it is important to continue research that clarifies mechanisms of risk and risk reduction in these populations. Understanding preconception, pregnancy and post‐natal factors associated with improved health outcomes is important for targeting and tailoring health care and support, and guiding intervention strategies that can alleviate intergenerational health risks.

## CONFLICTS OF INTEREST

Research grant (EIC181002) awarded by Guy's and St Thomas' Charity (London, UK) supported PD for a period of 4 months (part‐time) to conduct the work as part of this study.

## CONTRIBUTIONS

PD conceptualised the research under the supervision of JC, DB, SN and DP. PD was responsible for research investigation, project administration and research methodology (design and execution) under the supervision of JC, DB, SN and DP. JC, DB and PD acquired funding from Guy's and St Thomas' Charity. PD, SN and JC carried out formal analysis of the data. PD prepared the original manuscript draft. JC, DB, SN and DP contributed significantly to writing (review and editing) of manuscript. This article is the author(s) original work. This article has not received prior publication and is not under consideration for publication elsewhere. All authors have seen and approved the manuscript being submitted. JC is the data guarantor. Guy's and St Thomas' Charity do not have any permissions or influence over study design, methodology (including data collection), data analysis or manuscript preparation.

## Supporting information

**Table S1.** Missing Data Analysis: Comparison of Sociodemographic and Clinical Characteristics Among Women Classed as Completers and Non‐Completers.

## Data Availability

The data that support the findings of this study are available from the corresponding author (JC) upon reasonable request.
