# Supplementary material for: Infant feeding practices among macrosomic infants: A prospective cohort study
Source: Matern Child Nutr. 2021 Jun 1;17(4):e13222. doi: 10.1111/mcn.13222 (PMC8476408; doi:10.1111/mcn.13222)
Supplement: Supplementary file 1 — Table S1. Missing Data Analysis: Comparison of Sociodemographic and Clinical Characteristics Among Women Classed as Completers and Non‐Completers. [file MCN-17-e13222-s001.docx]

| Table S1 Missing Data Analysis: Comparison of Sociodemographic and Clinical Characteristics Among Women Classed as Completers and Non-Completers. | | | | |
| --- | --- | --- | --- | --- |
|  | Completer  (N = 328) | Non-Completer  (N = 85) | Statistic | p value  (α = .05) |
| Risk (N)  (High) | 89 (27.13%) | 39 (45.88%) | χ^2^ = 11.09 | .001* |
| Diabetes (N) (Yes)^#^ | 69 (21.04%) | 27 (31.76%) | χ^2^ = 9.16 | .010* |
| BMI (M) | 24.89 (5.07) | 26.91 (5.92) | t = 3.10 | .002* |
| BMI Category | N =318 | N = 82 |  |  |
| BMI ≤24.99 | 202 (61.59%) | 42 (49.41%) | χ^2^ = 14.24 | .003* |
| BMI ≥25.0 – 29.99 | 77 (23.48%) | 16 (18.81%) |  |  |
| BMI ≥30.0 | 39 (11.89%) | 24 (28.24%) |  |  |
| Age (M) | 35.08  (4.39) | 33.38 (5.04) | t = 3.09 | .002* |
| SES^‡^ (Mdn) | 4 (3 - 6) | 4 (2.5 - 5) | z = 2.30 | .021* |
| Parity (Primiparous) | 160 (48.78%) | 10 (11.76%) | χ^2^ = 2.39 | .122 |
| Gestation at Consent (M) | 226.80 (24.43) 32^+3^ | 224.61 (23.71) 32^+1^ | t = 0.81 | .420 |
| Macrosomic Infant (N) | 104 (31.71%) | 13 (15.29%) | χ^2^ = 8.96 | .003* |
| ^#^NB: All women with Type-1 diabetes were completers. Diabetic non-completers were all diagnosed with GDM. ^‡^Median rank and Inter Quartile Range (IQR) reported ^*^Significant at the α = 0.05 level. | | | | |
